# A case control study of occupation and cardiovascular disease risk in Japanese men and women

**DOI:** 10.1038/s41598-021-03410-9

**Published:** 2021-12-14

**Authors:** Kota Fukai, Yuko Furuya, Shoko Nakazawa, Noriko Kojimahara, Keika Hoshi, Akihiro Toyota, Masayuki Tatemichi

**Affiliations:** 1 Present Address: Department of Preventive Medicine, Tokai University School of Medicine, Isehara, Japan; 2 Department of Public Health, Shizuoka Graduate University of Public Health, Shizuoka, Japan; 3 Center for Public Health Informatics, National Institute of Public Health, Wako, Japan; 4 Department of Hygiene, School of Medicine, Kitasato University, Sagamihara, Japan; 5 Chugoku Rosai Hospital Research Center for the Promotion of Health and Employment Support, Japan Organization of Occupational Health and Safety, Hiroshima, Japan

**Keywords:** Epidemiology, Occupational health, Stroke

## Abstract

We aimed to investigate the risks of cardiovascular diseases associated with specific occupations, using a nation-wide, multicentre, hospital-based registry data from the Inpatient Clinico-Occupational Survey. The analysis included 539,110 controls (non-circulatory disease) and 23,792 cases (cerebral infarction, intracerebral/subarachnoid hemorrhage, acute myocardial infarction) aged ≥ 20 years who were initially hospitalized during 2005–2015. The participants’ occupational and clinical histories were collected by interviewers and medical doctors. Occupations were coded into 81 categories according to the Japanese standard occupation classification. Multivariable logistic regression analysis adjusted for age, admission year and hospital, smoking, alcohol consumption, hypertension, and shift-work was conducted by sex using general clerical workers as the reference. Increased risks of cerebral infarction, intracerebral hemorrhage, subarachnoid hemorrhage, and acute myocardial infarction, were observed in 15, 20, 25, and 1 occupation(s) in men, and 9, 2, 2, and 10 occupations in women. Motor vehicle drivers, food and drink preparatory workers, fishery workers, cargo workers, civil engineer workers, and other manual workers in men and other manual workers in women faced increased risks of all three stroke subtypes. Our findings demonstrate associations between specific occupations and the risk of cardiovascular disease incidence and suggest that the risk may vary by occupation.

## Introduction

The incidence of cardiovascular disease (CVD), including stroke and coronary heart disease (CHD), has exhibited a decreasing trend in Japan since the late twentieth century^1^. However, CVD remains associated with a high disease burden and major concerns in workers.

Previously, socio-epidemiological studies suggested that the occupational grade or class, a known social determinant of health, may also be a risk factor for CVD^2^. In the Whitehall study, Marmot et al. classified 17,530 British civil servants into four occupational grades (administrator, professional and executive, clerical, and other) and reported that the 10-year CHD mortality rate was three times higher in the lowest grade (other) relative to the highest grade (administrator)^3^. In Japan, Zaitsu et al. recently reported the risks of CHD and stroke incidence associated with broad occupational categories (manager, professional, service, and blue-collar) in combination with industrial clusters (white-collar, service, and blue-collar)^4^. Compared to the study in Britain^3^, the study in Japan identified a stronger pattern of CHD risk in higher occupational classes^4^. Other studies have also reported an association between CVD and occupational classes^5,6^, however, the results have been inconsistent. Therefore, it is difficult for clinicians to apply the findings in actual practice, even if they collected past occupational histories.

We hypothesized that there might be hidden hazards in various subcategorized occupations that may contribute to the risk of developing CVD. This research background suggests a need to focus on the specific occupation as epidemiological evidence rather than as a feature that can be represented by occupational grades or classes^7^. Generally, occupational classification schemes are not sufficiently detailed to enable a fine-scale analysis of associations with health measures^8^. One recent study demonstrated that the use of a detailed classification of occupations more accurately predicted health measures than did a broader classification or various combinations of socio-economic factors^7^. This may also be due to unknown occupational-related potential factors besides the effects of individual health status factors such as hypertension, smoking, or alcohol consumption, which are risk factors for CVD. In other words, it is important to examine the association between an individual’s detailed occupation and the risk of CVD onset from the viewpoint of occupational medicine. Roles may be different between men and women in the same occupation. To the best of our knowledge, no previously published study has explored this relationship. Therefore, we aimed to examine whether the incidence of CVD differs depending on the specific occupation among adults who participated in a nation-wide large-scale inpatient registry in Japan.

## Methods

### Study design and participants

This is a nation-wide multi-hospital-based case–control study. The present study was conducted using data from the Inpatient Clinico-Occupational Survey, which has been conducted by the Rosai Hospital Group since 1984. The details of this survey have been described elsewhere^4,9,10^. Briefly, the Inpatient Clinico-Occupational Survey collects clinical and occupational history information for all inpatients older than 15 years who were admitted to facilities belonging to the nation-wide Rosai Hospital Group (> 13,000 beds in 34 hospitals as in the year 2015) and remained alive at 24 h after admission. The clinical history data of each patient were combined with a summary of inpatient treatment recorded by physicians. Definitive diagnoses for admission were coded using the International Statistical Classification of Diseases and Related Health Problems, 10th Revision (ICD-10)^11^. Lifestyle habits (e.g., smoking and alcohol consumption) and history of hypertension were also included in the clinical history. All information was registered in the Inpatient Clinico-Occupational Database by the health information manager and a trained occupational history surveyor at each hospital. Data were collected at each admission for patients who were hospitalized multiple times during the study period. The clinical and occupational history registry rates were nearly 100% and 66%, respectively.

For the present study, we used data collected during 2005–2015, when the clinical and occupational data of 829,926 participants were subjected to analysis. For patients who were hospitalized multiple times, only the first hospitalization data were used. We excluded participants younger than 20 years (n = 18,094); those whose occupation were unclassifiable (n = 303); those for whom were unemployed as the longest-held occupation (n = 77,895), student (n = 18,993), homemaker (n = 118,973). Furthermore, we excluded those admitted for diseases of the circulatory system except CVD (n = 32,825). Finally, 562,902 participants remained for analysis.

This study was performed according to the tenets of the Declaration of Helsinki. Written informed consent was obtained from each patient prior to completion of all the questionnaires. Access to the dataset was provided under a research agreement between the study authors and the Japan Organization of Occupational Health and Safety. This study was approved by the Research Ethics Committees of Tokai University School of Medicine, Kanagawa, Japan (Protocol Number 18R-309) and the Japan Organization of Occupational Health and Safety (Protocol Number R1-006).

### Cases and controls

CVD cases were defined as patients with a diagnosis at admission of cerebral infarction (ICD-10, I63; n = 12,826), intracerebral haemorrhage (ICD-10, I61; n = 4905), subarachnoid haemorrhage (ICD-10, I60; n = 1641), and acute myocardial infarction (ICD-10, I21; n = 4420). Consistent with the methodology used in previous studies^12^, the controls were defined as patients who were admitted at the same hospital and during the same period for reasons other than diseases of the circulatory system (ICD-10, I00 to I99 except for I21, I60, I61, I63; n = 65,591). The final analysis included 23,792 cases and 539,110 controls. The participant flowchart is outlined in S1 Fig.

### Occupation assessment

The participants were interviewed regarding their current and three previous occupations, as well as the duration of each. Occupational history information was coded according to the Japan Standard Occupational Classification, which was published by the Japanese Ministry of Internal Affairs and Communications^13^ by a trained occupational history surveyor. To accommodate the revision of the Japan Standard Occupational Classification which was published by the government during the survey, the Japan Organisation of Occupational Health and Safety created an original unified code of 81 occupations that would cover the revision. This unified classification is roughly compliant with the sub-major (two-digit codes, 43 groups) and minor (three-digit codes, 130 groups) categories of the International Standard Classification of Occupations 2008 (ISCO-08)^14^. For each participant, the longest-held of the four most recent occupations was adopted as the exposure variable. Information about shift-work history was also collected. The list of occupations and numbers of controls and cases in detail are presented in S1 Table.

### Statistical analyses

The odds ratios (ORs) and 95% confidence intervals (95% CIs) of the incidence of CVD (cerebral infarction, intracerebral hemorrhage, subarachnoid hemorrhage, and acute myocardial infarction) were estimated using multivariable logistic regression analysis. We used general clerical workers as the reference group in all analyses because it was the most frequent category for both men and women and was considered to have less effect of various occupational hazards. Lifestyle habits (e.g., smoking and alcohol consumption) and history of hypertension were also included in the clinical history. Model 1 was the unadjusted model. Model 2 was created by adjusting for age (in years, continuous), admission date (in years, categorical), and admitting hospital (34 hospitals, categorical). We the adjusted admission date to explore potential heterogeneity introduced by changes in the environment within the occupation and by secular changes in diagnostic practices or treatment. Also, we adjusted the admission hospital to account for differences by region, despite the same occupation, such as rural and urban areas, since this database was nationwide database. Model 3 was further adjusted for smoking (never smoker, former smoker, current smoker), alcohol consumption (non-drinker, former drinker, current drinker), and hypertension (yes/no). In Japan, hypertension has a high prevalence and the outcome (CVD) is not only caused by the exposure (occupation). Since hypertension is both associated with occupation and CVD, we adjusted hypertension in model 3 as a potential cofounder. Model 4 was further adjusted for shift-work (yes/no). No missing values for confounders in the dataset for this analysis existed due to a standard protocol by trained interviewers.

While CVD is chronically affected by the cardiovascular system, it is a sudden-onset disease, and to account for acute effects, we conducted a sensitivity analysis using the current occupation as an exposure variable. A two-sided *p* value < 0.05 was considered statistically significant. All analyses were performed using the Statistical Analysis System (SAS) Software version 9.4 (SAS Institute, Cary, NC, USA).

## Results

The characteristics of the cases and controls are presented in Table 1. Notably, the distributions of age and smoking status differed between these groups. Cases of subarachnoid hemorrhage included younger patients and tended to include large proportions of current smokers and alcohol drinkers.

**Table 1:** Characteristics of participants.

	Male		Female	
	Controls, N (%)	Cases, N (%)	Controls, N (%)	Cases, N (%)
N	336,410	17,884	202,694	5909
**Age, years**				
< 65 years	187,138 (55.6%)	7821 (43.7%)	138,265 (68.2%)	2055 (34.8%)
≥ 65 years	149,272 (44.4%)	10,063 (56.3%)	64,429 (31.8%)	3854 (65.2%)
**Smoking status**				
None	73,082 (21.7%)	3864 (21.6%)	149,734 (73.9%)	4819 (81.6%)
Past	144,727 (43.0%)	6953 (38.9%)	27,506 (13.6%)	392 (6.6%)
Current	118,601 (35.3%)	7067 (39.5%)	25,454 (12.6%)	698 (11.8%)
**Alcohol consumption**				
None	128,971 (38.3%)	6606 (36.9%)	156,836 (77.4%)	4994 (84.5%)
Past	39,196 (11.7%)	2243 (12.5%)	16,233 (8.0%)	246 (4.2%)
Current	168,243 (50.0%)	9035 (50.5%)	29,625 (14.6%)	669 (11.3%)
Hypertension, yes	93,283 (27.7%)	7423 (41.5%)	38,867 (19.2%)	2459 (41.6%)
Shift-work, yes	19,553 (5.8%)	722 (4.0%)	14,697 (7.3%)	195 (3.3%)

**Figure 1:**
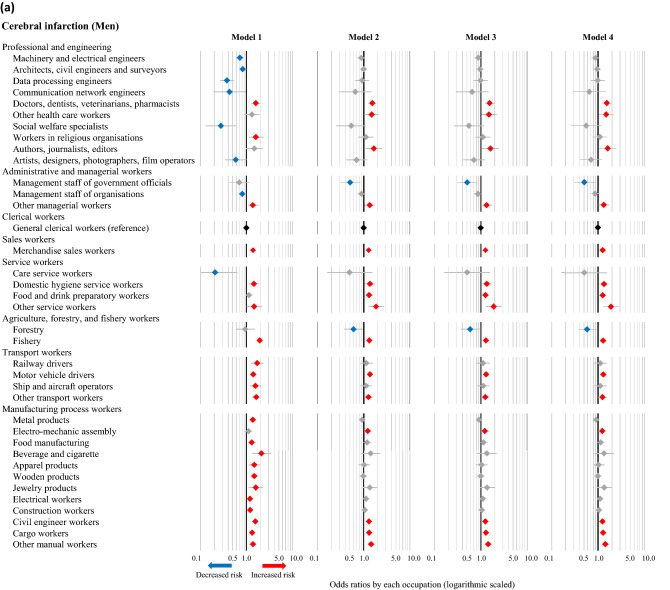

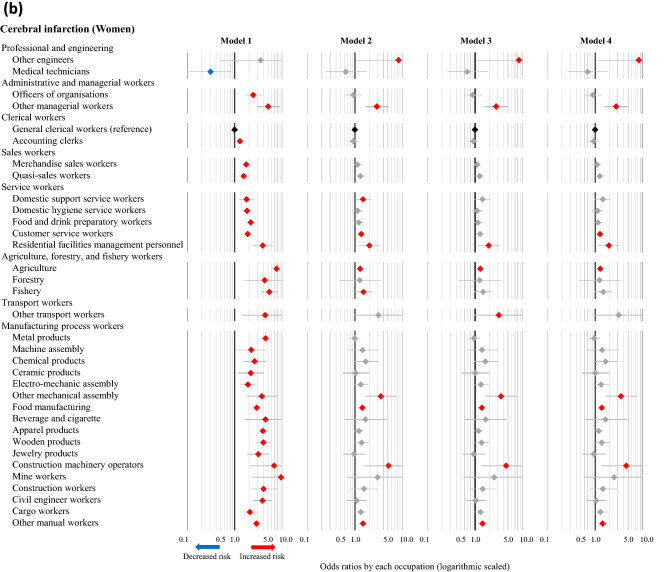
Odds ratios of cerebral infarction associated with occupations among men (**a**) and women (**b**) based on different models. The odds ratios (symbols) and 95% confidence intervals (bars) were estimated using univariate and multivariable logistic regression analyses, with general clerical workers as the reference. Red and blue symbols indicate statistically significant increases and decreases in risk, respectively. Model 1: Unadjusted. Model 2: Adjusted for age, admission date, and hospital. Model 3: Adjusted for the factors in Model 2 plus smoking, alcohol consumption, and hypertension. Model 4: Adjusted for the factors in Model 3 plus shift-work.

Cerebral infarction accounted for the largest number of cases among both men and women, and the ORs of various occupations for the incidence of cerebral infarction were calculated using each model. The statistically significant results corresponding to occupations resulting from any of the four models are described in Fig. 1a (men) and Fig. 1b (women), and the full results are available in S2 Table. In the fully adjusted model (Model 4), high ORs of cerebral infarction were observed in 15 occupations among men (doctors, dentists, veterinarians, pharmacists; other health care workers; authors, journalists, editors; other managerial workers; merchandise sales workers; domestic hygiene service workers; food and drink preparatory workers; other service workers; fishery; motor vehicle drivers; other transport workers; electro-mechanic assembly workers; civil engineer workers; cargo workers; and other manual workers), as well as 9 occupations among women (other engineers; other managerial workers; customer service workers; residential facilities management personnel; agriculture; other mechanical assembly; food manufacturing; construction machinery operators; and other manual workers). Low ORs of cerebral infarction were observed in management staff of government officials and forestry among men, and none among women. The results of the unadjusted model (Model 1) differed somewhat from those of the other models. After adjusting for age (Model 2), however, the results remained consistent even with further adjustments (e.g., admission date, hospital, smoking, drinking, hypertension, and shift-work). The full results of cases of intracerebral hemorrhage, subarachnoid hemorrhage, and acute myocardial infarction are available in S3–S5 Tables. These results were similar to those of cases of cerebral infarction, although smoking and alcohol consumption also appeared to affect the incidence of subarachnoid hemorrhage and acute myocardial infarction. Shift-work had no apparent effect on the associations of occupations with CVD.

**Figure 2:**
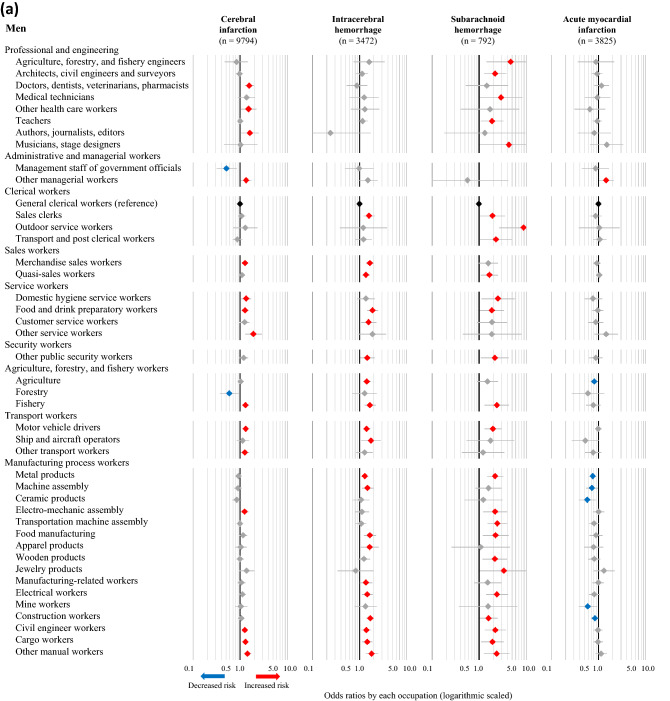

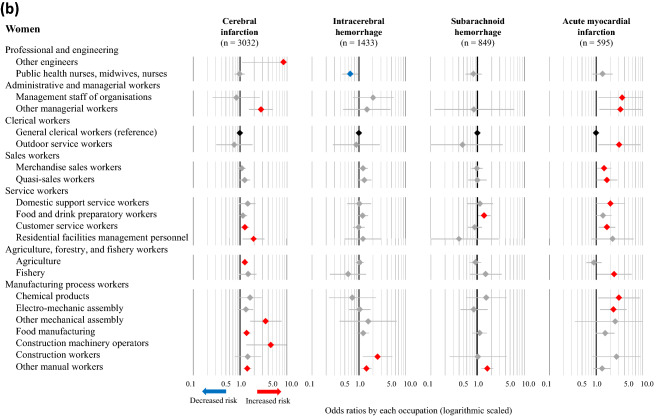
Odds ratios for CVD associated with occupations among men (**a**) and women (**b**). The odds ratios (symbols) and 95% confidence intervals (bars) were estimated using multivariable logistic regression analysis, with general clerical workers as the reference. The analysis was adjusted for age, admission date, hospital, smoking, alcohol consumption, hypertension, and shift-work. Red and blue symbols indicate statistically significant increases and decreases in risk, respectively.

The fully adjusted ORs (Model 4) for cerebral infarction, intracerebral hemorrhage, subarachnoid hemorrhage, and acute myocardial infarction in association with various occupations were also calculated, using general clerical workers as the reference group. The statistically significant results corresponding to specific occupations in any of the four disease categories are presented in Fig. 2a (men) and Fig. 2b (women), while the full results are available in S2–S5 Tables. The number of categories that were significantly associated with increased risks of cerebral infarction, intracerebral hemorrhage, subarachnoid hemorrhage, and acute myocardial infarction were, 15, 20, 25, and one occupation(s) among men, respectively, while two, zero, zero, and six occupations were associated with decreased risks of the respective conditions. Moreover, among men, increased risks of all three subtypes of stroke were observed in food and drink preparatory workers, fishery, motor vehicle drivers, civil engineer workers, cargo workers, and other manual workers. Among women, nine, two, two, and ten occupations were associated with a significantly increased risk of cerebral infarction, intracerebral hemorrhage, subarachnoid hemorrhage, and acute myocardial infarction, respectively, while one, zero, zero, and zero occupations were associated with decreased risks of the respective conditions. Moreover, female participants classified as other manual workers had increased risks of all three subtypes of stroke. Female food and drink preparatory workers also exhibited an increasing trend in the risk of all three subtypes of stroke, although these associations were not statistically significant.

**Figure 3:**
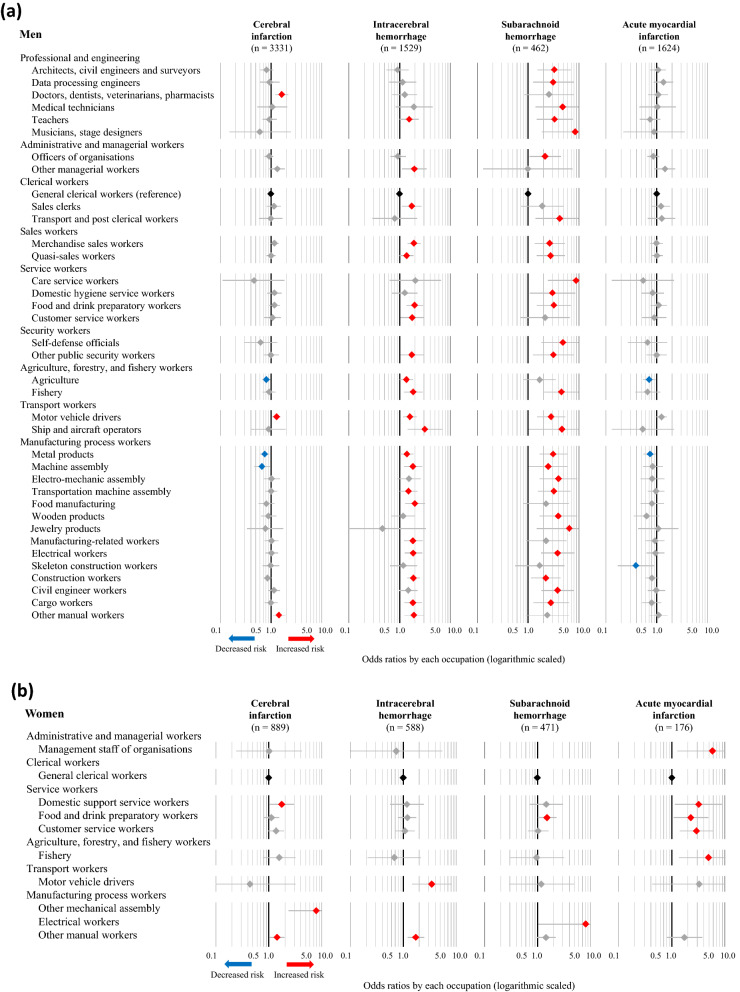
Odds ratios for CVD associated with occupations among men (**a**) and women (**b**) whose current occupation was also the longest-held occupation. The odds ratios (symbols) and 95% confidence intervals (bars) were estimated using multivariable logistic regression analysis, with general clerical workers as the reference. The analysis was adjusted for age, admission date, hospital, smoking, alcohol consumption, hypertension, and shift-work. Red and blue symbols indicate statistically significant increases and decreases in risk, respectively.

The results of the sensitivity analysis, which included only participants whose current occupation was also the longest-held occupation, are presented in Fig. 3a (men) and Fig. 3b (women) as fully adjusted ORs (Model 4) of the incidence of CVD. Among men, two, 17, and 19 occupations were associated with an increased risk of cerebral infarction, intracerebral hemorrhage, and subarachnoid hemorrhage, respectively, to the same degree observed in the full population analysis, while two occupations were associated with a decreased risk. Consistent with the full analysis, male motor vehicle drivers continued to face increased risks of all three subtypes of stroke. Among women, two, one, and four occupations were associated with an increased risk of cerebral infarction, intracerebral hemorrhage, and acute myocardial infarction, respectively, to the same degree observed in the full population analysis.

## Discussion

In this study, based on a data set from a nation-wide large-scale inpatient survey in Japan, we explored the potential associations between specific occupations and CVD. Although some previous studies have demonstrated associations between occupational classes or grades and CVD^3,4,15–17^, ours is one of the first to provide descriptive results associated with specific occupations. Interestingly, there were clear differences in the association of specific occupations with a risk for CVD between men and women, cerebral versus coronary, and between cerebral infarction and hemorrhage. There was one specific occupation that increased the risk for CHD in men, but ten occupations showed a significantly increased risk in women. On the other hand, men had many occupations that increased the risk for cerebrovascular diseases, but women had few.

Among men, only other management workers increased the risk for CHD. On the other hand, motor vehicle drivers, food and drink preparatory workers, and other manual workers exhibited a robust increase in the risk of all three subtypes of stroke when compared with the reference group of general clerical workers. However, in our sensitivity analysis (Fig. 3a), only doctors, motor vehicle drivers, and other manual workers showed a slightly increased risk for cerebral infarction. While quasi-sales workers (i.e. other sales workers), food and drink preparatory workers, fishery workers, and manufacturing process workers (e.g., metal producers, skeleton construction workers, civil engineers, and cargo workers) faced increased risks of both intracerebral and subarachnoid hemorrhage, but not cerebral infarction. Hypertension plays a pivotal role in stroke, particularly hemorrhage^18^, although we conducted model-based adjustment for hypertension based on information of the present illness. We consider that these specific occupations with an increased risk for hemorrhage may temporarily and markedly elevate blood pressures in workers by mental or physical stresses. In addition, the ‘physical activity paradox’ is recently emphasized, and occupational physical activity (OPA) is considered to play an important role in CVD^19^. According to Steeves’s classifications^20^, the occupations that were found to be associated with intracerebral hemorrhage were classified as occupations with high OPA levels. The occupations with high odds ratios of intracerebral haemorrhage such as agriculture, forestry, and fishery engineers (OR = 4.72), outdoor service workers (OR = 8.86), communication workers (OR = 3.60) are considered to be high OPA level. Further investigation of OPA levels and CVD risk is needed.

In women, there were significant occupations associated with an increased risk of CHD, such as management staff of organizations, domestic support service workers, customer service workers, and fishery workers. These findings were different from those in men suggesting that it might be more mentally stressful for women than men, resulting in the development of coronary arterial atherosclerosis. However, the associations between specific occupations and cerebral hemorrhages were less clear. Three types of manufacturing process workers (food manufacturing, construction machinery operators, and other mechanical assembly workers) had a strongly increased risk of stroke. Orthostatic hypotension is an important risk factor for stroke^21^. Thus, among workers with these occupations, they stand all day, and chronic orthostatic hypotension might be a risk factor. Other engineers and other managerial workers also showed an increased risk for stroke. However, this classification was not clear, and the reason was unknown.

Motor vehicle drivers and food and drink preparatory workers exhibited a robust increase in the risk of all three subtypes of stroke. As for other possibilities, we may point out the risk of passive smoking. This is a major public health issue that is considered a risk factor for CVD^22–24^. In Japan, the Health Promotion Law enacted in 2003 obligated administrators of public facilities to make efforts to prevent passive smoking. However, restaurants and taxicabs were the least developed areas for passive smoking prevention. Until the late 2000s, Japanese taxi drivers were exposed to second-hand cigarette smoke due to passengers^25^. Many restaurants that employ food and drink preparatory workers do not impose restrictions on smoking, which was allowed in 95.9% of the customer areas and 85.0% of the employee work areas in restaurants in 2007^26^. According to the Japanese National Health and Nutrition Survey in 2013, 50.9% of people were exposed to second-hand smoke at restaurants^27^. The evidence suggests that second-hand smoke may have contributed to the increased risk of CVD among subjects employed in workplaces without smoking restrictions.

Although it is difficult to compare our findings with those of previous studies that focused on occupational classes or grades, our results generally appear to be consistent with those of earlier analyses. Particularly, socioeconomically advantaged groups tend to have a lower risk of CVD^6^, whereas so-called manual workers have a higher risk^16,28^. Our results demonstrate that several occupations in the categories of manufacturing process, service, and sales were associated with a high risk of CVD even after adjusting for age, smoking and alcohol consumption habits, hypertension, and shift work. Thus, our results indicate that information on the occupation of patients is important for physicians to prevent the onset of CVD.

One strength of this study is the use of an extensive set of detailed information about previous and current occupations which was collected by professionally trained investigators. An analysis of the relationships between occupations and CVD risk requires a large population for which specific occupation data are available. Although hypertension and smoking are the risk factors most strongly associated with CVD^29^, an assessment focused on the occupational histories of participants in a clinical population at risk of CVD is important from the viewpoint of occupational medicine. We noted that in our survey, the association between occupation and CVD remained even after other factors were adjusted, suggesting the involvement of hidden occupation-related factors.

Our study had limitations. First, the study may have been subject to selection bias regarding the controls (i.e., Berkson’s bias)^30^. The hospital admission probability is defined as the probability that the members of a community group will be admitted to the hospital^31^, and we selected controls from among patients admitted to the same hospitals as the cases. The distribution of the occupational profiles of our controls were nationally representative^4,9^. Yet, bias towards or away from null association may exist in the selection of hospital controls, who may be more biased toward risk factors for being hospitalized for any disease than the general population. Second, data regarding other clinical risk factors for CVD, such as blood pressure, total cholesterol, and glucose intolerance, were unavailable. However, we used self-reported data adjusted for a history of hypertension. Third, other socioeconomic covariates such as working hours or income which were examined in other relevant studies^6,32^ were not assessed due to the lack of data. Employees who worked long hours had a higher risk of stroke than those working standard hours; the association with CHD was weaker^32^. Future studies should consider the effects of long working hours on the difference of onset of stroke and CHD. No strong association between the income level and CVD risk has been observed in Japan^6^. For available factors, we adjusted shift work as a work-related factor and achieved consistent results. Fourth, we considered only the longest-held occupation as the risk factor, and we did not consider the impact of a change nor sequence in employment. There is a possibility that they may have changed jobs or become unemployed depending on their health conditions. Further assessment is needed to address this variable.

In conclusion, the present study has clearly demonstrated the difference between long-held specific occupations and the risk of CVD. From the viewpoint of occupational medicine, our results may be valuable to investigate key factors among these potentially high-risk occupations in terms of the risk of CVD.

## Supplementary Information

Supplementary Figure S1.

Supplementary Table S1.

Supplementary Table S2.

Supplementary Table S3.

Supplementary Table S4.

Supplementary Table S5.

## Data Availability

The datasets are not publicly available due to restrictions used under the license for the current study. There are available on reasonable request from the corresponding author.
