# Supplementary figures and images for: A case control study of occupation and cardiovascular disease risk in Japanese men and women
Source: Sci Rep. 2021 Dec 14;11:23983. doi: 10.1038/s41598-021-03410-9 (PMC8671491; doi:10.1038/s41598-021-03410-9)

**S1 Fig. Flow diagram of the inclusion and exclusion of participants.**

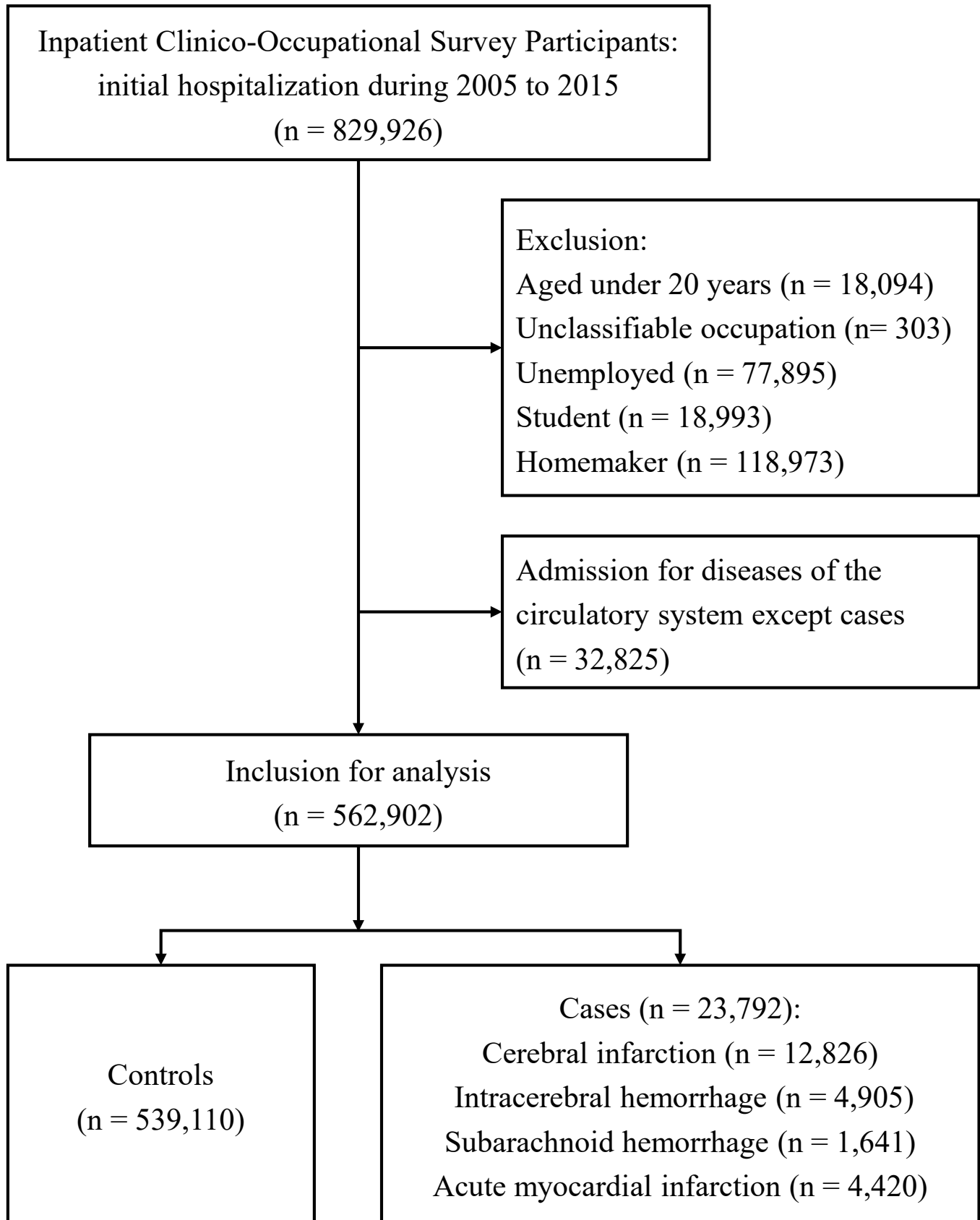

Supplement: Supplementary file 1 — Supplementary Figure S1. [file 41598_2021_3410_MOESM1_ESM.pdf]
