# Supplementary material for: A case control study of occupation and cardiovascular disease risk in Japanese men and women
Source: Sci Rep. 2021 Dec 14;11:23983. doi: 10.1038/s41598-021-03410-9 (PMC8671491; doi:10.1038/s41598-021-03410-9)
Supplement: Supplementary file 2 — Supplementary Table S1. [file 41598_2021_3410_MOESM2_ESM.pdf]

S1 Table. The occupations and numbers of controls and cases in the study.

	Men					Women				
	Control	Cerebral infarction	Intracerebral hemorrhage	Subarachnoid hemorrhage	Acute myocardial infarction	Control	Cerebral infarction	Intracerebral hemorrhage	Subarachnoid hemorrhage	Acute myocardial infarction
	N	N	N	N	N	N	N	N	N	N
All	354296	9794	3472	792	3825	202697	3032	1433	849	595
Professional and engineering										
Researchers	489	9	5	1	4	152	2	1	1	0
Agriculture, forestry, and fishery engineers	455	13	7	3	5	51	0	0	0	0
Food engineers	188	5	2	0	1	110	0	1	0	0
Machinery and electrical engineers	7096	123	43	12	65	289	2	0	0	0
Industrial engineers	2132	46	7	4	15	284	1	1	0	0
Other manufacturing engineers	619	10	3	0	3	35	0	0	0	0
Architects, civil engineers, surveyors	7837	155	57	21	79	319	1	1	0	0
Data processing engineers	3729	34	14	7	34	1012	2	0	2	0
Communication network engineers	571	6	1	0	4	55	0	0	0	0
Other engineers	547	7	3	0	2	38	1	0	0	0
Doctors, dentists, veterinarians, pharmacists	2583	96	16	4	34	1390	10	5	1	2
Public health nurses, midwives, nurses	459	6	3	2	4	10897	81	33	31	22
Medical technicians	1095	25	8	4	10	1732	4	4	1	2
Other health care workers	941	29	8	2	7	4079	26	15	10	4
Social welfare specialists	1014	7	7	0	4	6502	44	21	16	6
Legal workers	447	7	2	0	5	103	0	0	0	0
Finance and insurance professionals	902	16	9	1	9	184	1	0	0	0
Teachers	9044	241	83	19	96	6865	58	41	25	14
Workers in religious organisations	834	31	8	2	11	123	2	3	1	0
Authors, journalists, editors	655	23	1	1	6	255	1	0	2	0
Artists, designers, photographers, film operators	1003	14	10	3	14	718	3	2	1	0
Musicians, stage designers	413	6	0	2	6	213	0	2	0	0
Other specialist professionals	2734	53	17	5	29	3268	29	18	8	6
Administrative and managerial workers										
Management staff of government officials	952	16	8	0	9	93	0	0	0	1
Officers of organisations	11245	334	87	22	137	1363	25	19	4	6
Management staff of organisations	6346	123	35	10	80	360	3	4	0	3
Other managerial workers	1953	63	19	1	34	341	13	3	1	4
Clerical workers										
General clerical workers	31404	745	229	37	345	37684	282	169	125	54
Accounting clerks	4324	92	29	1	43	8177	79	42	22	18
Production-related clerical workers	3725	70	19	6	43	1226	14	3	6	1
Sales clerks	6493	130	69	15	54	3812	20	8	4	3
Outdoor service workers	329	12	3	3	4	521	5	3	1	4
Transport and post clerical workers	2739	77	29	8	38	704	8	3	1	2
Office appliance operators	305	4	0	1	3	953	5	1	3	2
Sales workers										
Merchandise sales workers	15240	495	190	31	172	21364	279	160	87	73
Quasi-sales workers	25645	563	229	55	300	5593	65	38	22	20
Service workers										
Domestic support service workers	135	1	0	0	0	2182	29	12	10	9
Care service workers	596	3	3	3	2	2710	14	12	7	3
Domestic hygiene service workers	2264	77	23	7	22	4960	67	33	24	14
Food and drink preparatory workers	6046	161	77	16	66	9378	153	84	64	38
Customer service workers	3362	76	32	9	32	10518	147	65	44	43
Residential facilities management personnel	834	27	8	1	9	517	15	5	1	4
Other service workers	790	27	9	2	12	1137	8	4	2	2
Security workers										
Self-defense officials	2912	61	14	9	28	97	0	0	0	0
Judicial police staff	2471	55	17	3	21	120	1	0	0	0
Other public security workers	4003	105	37	11	41	202	0	1	1	0
Agriculture, forestry, and fishery workers										
Agriculture	13906	681	238	27	180	12581	719	246	98	82
Forestry	848	19	12	0	6	124	4	1	0	1
Fishery	4564	203	69	16	35	646	26	5	7	6
Transport workers										
Railway drivers	1060	42	13	0	14	4	0	0	0	0
Motor vehicle drivers	22049	723	236	60	266	622	7	6	4	1
Ship and aircraft operators	1350	49	19	3	9	13	0	0	0	0
Other transport workers	2653	101	27	4	27	91	3	0	0	0
Communication workers	487	6	4	2	4	1136	10	5	4	2
Manufacturing process workers										
Metal products	23588	758	286	77	252	2548	85	41	15	14
Machine assembly	5877	126	64	13	53	477	8	2	1	0
Chemical products	5036	139	47	10	47	663	13	3	4	4
Ceramic products	3181	80	27	5	22	614	10	5	0	1
Electro-mechanic assembly	4949	128	38	15	55	2397	34	16	9	11
Transportation machine assembly	8600	209	69	31	80	635	3	5	5	2
Other mechanical assembly	572	17	3	1	11	250	7	2	0	1
Food manufacturing	4043	123	52	13	41	6259	137	66	40	27
Beverage and cigarette	371	18	6	0	4	119	4	1	1	0
Apparel products	1499	52	22	2	19	4496	133	37	28	24
Wooden products	4794	165	59	15	53	1091	33	16	1	4
Printing and bookbinding	1561	33	16	1	14	546	7	2	2	1
Rubber and plastic products	1513	37	15	3	20	625	8	5	3	0
Jewelry products	892	33	5	3	15	593	14	6	4	1
Manufacturing-related workers	4871	116	48	11	58	980	5	6	2	1
Construction machinery operators	5532	149	36	6	69	59	3	0	0	0
Electrical workers	6575	186	73	21	62	62	0	0	1	0
Mine workers	2111	47	14	2	22	28	2	1	1	1
Skeleton construction workers	3424	85	17	8	29	42	0	0	0	0
Construction workers	18707	530	252	42	184	329	10	8	2	3
Civil engineer workers	7854	284	91	24	89	659	19	11	2	2
Cargo workers	7589	236	80	20	86	2638	41	24	18	10
Other manual workers	4340	140	54	13	57	8684	187	96	69	36
