# Supplementary material for: A case control study of occupation and cardiovascular disease risk in Japanese men and women
Source: Sci Rep. 2021 Dec 14;11:23983. doi: 10.1038/s41598-021-03410-9 (PMC8671491; doi:10.1038/s41598-021-03410-9)
Supplement: Supplementary file 3 — Supplementary Table S2. [file 41598_2021_3410_MOESM3_ESM.pdf]

S2 Table. Odds ratios for cerebral infarction by occupations among men and women.

	Model 1	Model 2	Model 3	Model 4
Men				
Professional and engineering				
Researchers	0.77 (0.40, 1.50)	0.88 (0.45, 1.72)	0.90 (0.46, 1.76)	0.90 (0.46, 1.76)
Agriculture, forestry, and fishery engineers	1.23 (0.70, 2.14)	0.79 (0.45, 1.39)	0.83 (0.47, 1.45)	0.83 (0.47, 1.45)
Food engineers	1.12 (0.46, 2.73)	1.34 (0.55, 3.30)	1.40 (0.57, 3.45)	1.40 (0.57, 3.45)
Machinery and electrical engineers	0.72 (0.60, 0.88)	0.88 (0.72, 1.06)	0.89 (0.73, 1.08)	0.89 (0.73, 1.08)
Industrial engineers	0.90 (0.67, 1.22)	0.99 (0.73, 1.34)	0.99 (0.73, 1.35)	0.99 (0.73, 1.35)
Other manufacturing engineers	0.67 (0.36, 1.00)	0.84 (0.44, 2.00)	0.86 (0.45, 2.00)	0.86 (0.45, 2.00)
Architects, civil engineers, surveyors	0.83 (0.70, 0.99)	0.99 (0.83, 1.18)	0.97 (0.81, 1.15)	0.97 (0.81, 1.15)
Data processing engineers	0.38 (0.27, 0.53)	0.93 (0.65, 1.31)	0.99 (0.70, 1.40)	0.99 (0.70, 1.40)
Communication network engineers	0.43 (0.19, 0.97)	0.65 (0.29, 1.47)	0.65 (0.29, 1.48)	0.65 (0.29, 1.48)
Other engineers	0.53 (0.25, 1.12)	0.74 (0.35, 1.57)	0.73 (0.34, 1.55)	0.73 (0.34, 1.55)
Doctors, dentists, veterinarians, pharmacists	1.59 (1.28, 1.98)	1.53 (1.23, 1.91)	1.56 (1.25, 1.95)	1.56 (1.25, 1.95)
Public health nurses, midwives, nurses	0.55 (0.24, 1.22)	0.93 (0.41, 2.10)	0.93 (0.41, 2.10)	0.94 (0.42, 2.14)
Medical technicians	0.96 (0.64, 1.44)	1.30 (0.86, 1.96)	1.37 (0.91, 2.07)	1.37 (0.91, 2.07)
Other health care workers	1.31 (0.90, 1.91)	1.49 (1.01, 2.18)	1.51 (1.03, 2.22)	1.51 (1.03, 2.22)
Social welfare specialists	0.28 (0.13, 0.60)	0.53 (0.25, 1.13)	0.55 (0.26, 1.18)	0.56 (0.26, 1.18)
Legal workers	0.65 (0.31, 1.38)	0.54 (0.25, 1.15)	0.56 (0.26, 1.19)	0.56 (0.26, 1.19)
Finance and insurance professionals	0.74 (0.45, 1.23)	0.72 (0.44, 1.20)	0.71 (0.43, 1.18)	0.71 (0.43, 1.18)
Teachers	1.13 (0.97, 1.31)	0.98 (0.85, 1.14)	1.00 (0.86, 1.17)	1.00 (0.86, 1.17)
Workers in religious organisations	1.60 (1.11, 2.31)	1.11 (0.76, 1.61)	1.11 (0.76, 1.61)	1.11 (0.76, 1.61)
Authors, journalists, editors	1.49 (0.97, 2.27)	1.65 (1.07, 2.54)	1.62 (1.05, 2.49)	1.62 (1.05, 2.49)
Artists, designers, photographers, film operators	0.59 (0.34, 1.00)	0.70 (0.41, 1.20)	0.71 (0.42, 1.22)	0.71 (0.41, 1.22)
Musicians, stage designers	0.61 (0.27, 1.36)	0.99 (0.44, 2.24)	1.03 (0.45, 2.33)	1.03 (0.45, 2.32)
Other specialist professionals	0.81 (0.61, 1.08)	1.02 (0.77, 1.36)	1.05 (0.79, 1.40)	1.05 (0.79, 1.40)
Administrative and managerial workers				
Management staff of government officials	0.70 (0.43, 1.16)	0.51 (0.31, 0.85)	0.51 (0.31, 0.85)	0.51 (0.31, 0.85)
Officers of organisations	1.26 (1.11, 1.44)	1.04 (0.91, 1.19)	1.02 (0.89, 1.17)	1.02 (0.89, 1.17)
Management staff of organisations	0.81 (0.67, 0.99)	0.89 (0.73, 1.08)	0.87 (0.72, 1.06)	0.87 (0.72, 1.06)
Other managerial workers	1.38 (1.07, 1.80)	1.35 (1.04, 1.76)	1.34 (1.02, 1.75)	1.34 (1.02, 1.75)
Clerical workers				
General clerical workers	reference	reference	reference	reference
Accounting clerks	0.89 (0.72, 1.11)	0.91 (0.73, 1.14)	0.91 (0.73, 1.13)	0.91 (0.73, 1.14)
Production-related clerical workers	0.79 (0.61, 1.01)	0.84 (0.66, 1.08)	0.83 (0.64, 1.06)	0.83 (0.64, 1.06)
Sales clerks	0.84 (0.70, 1.02)	1.09 (0.90, 1.32)	1.07 (0.89, 1.30)	1.07 (0.89, 1.30)
Outdoor service workers	1.58 (0.88, 2.82)	1.30 (0.72, 2.36)	1.28 (0.71, 2.33)	1.28 (0.71, 2.33)
Transport and post clerical workers	1.20 (0.95, 1.52)	0.88 (0.69, 1.12)	0.88 (0.69, 1.12)	0.88 (0.69, 1.12)
Office appliance operators	0.54 (0.20, 1.00)	1.03 (0.38, 3.00)	1.11 (0.41, 3.00)	1.11 (0.41, 3.00)
Sales workers				
Merchandise sales workers	1.39 (1.24, 1.56)	1.28 (1.14, 1.44)	1.27 (1.13, 1.43)	1.27 (1.13, 1.43)
Quasi-sales workers	0.93 (0.83, 1.04)	1.10 (0.99, 1.24)	1.09 (0.97, 1.22)	1.09 (0.97, 1.22)
Service workers				
Domestic support service workers	0.30 (0.04, 2.00)	0.64 (0.09, 5.00)	0.64 (0.09, 5.00)	0.65 (0.09, 5.00)
Care service workers	0.21 (0.07, 0.64)	0.49 (0.16, 1.52)	0.51 (0.16, 1.59)	0.51 (0.16, 1.61)
Domestic hygiene service workers	1.45 (1.15, 1.85)	1.36 (1.06, 1.73)	1.35 (1.06, 1.72)	1.35 (1.06, 1.72)
Food and drink preparatory workers	1.13 (0.95, 1.35)	1.31 (1.10, 1.56)	1.27 (1.06, 1.51)	1.27 (1.07, 1.52)
Customer service workers	0.95 (0.75, 1.21)	1.25 (0.98, 1.60)	1.24 (0.97, 1.58)	1.24 (0.98, 1.59)
Residential facilities management personnel	1.38 (0.93, 2.04)	1.45 (0.98, 2.16)	1.40 (0.94, 2.09)	1.40 (0.94, 2.09)
Other service workers	1.47 (1.00, 2.18)	1.87 (1.26, 2.79)	1.91 (1.28, 2.85)	1.91 (1.28, 2.85)
Security workers				
Self-defense officials	0.88 (0.67, 1.14)	0.81 (0.62, 1.07)	0.82 (0.63, 1.07)	0.82 (0.63, 1.07)
Judicial police staff	0.93 (0.71, 1.23)	1.10 (0.83, 1.45)	1.11 (0.83, 1.46)	1.11 (0.84, 1.47)
Other public security workers	1.11 (0.90, 1.37)	1.21 (0.98, 1.50)	1.18 (0.96, 1.46)	1.19 (0.96, 1.47)
Agriculture, forestry, and fishery workers				
Agriculture	2.15 (1.93, 2.39)	1.02 (0.91, 1.13)	1.03 (0.92, 1.15)	1.03 (0.92, 1.15)
Forestry	0.94 (0.60, 1.50)	0.60 (0.37, 0.95)	0.59 (0.37, 0.94)	0.59 (0.37, 0.94)
Fishery	1.93 (1.65, 2.26)	1.32 (1.12, 1.55)	1.30 (1.10, 1.53)	1.30 (1.10, 1.53)
Transport workers				
Railway drivers	1.71 (1.24, 2.00)	1.14 (0.83, 2.00)	1.13 (0.81, 2.00)	1.13 (0.82, 2.00)
Motor vehicle drivers	1.40 (1.27, 1.56)	1.37 (1.23, 1.52)	1.31 (1.18, 1.46)	1.32 (1.18, 1.46)
Ship and aircraft operators	1.56 (1.16, 2.09)	1.12 (0.83, 1.50)	1.13 (0.84, 1.52)	1.13 (0.84, 1.53)
Other transport workers	1.63 (1.32, 2.02)	1.26 (1.02, 1.57)	1.25 (1.01, 1.55)	1.26 (1.01, 1.56)
Communication workers	0.51 (0.23, 1.15)	0.48 (0.21, 1.08)	0.49 (0.22, 1.10)	0.49 (0.22, 1.10)
Manufacturing process workers				

Metal products	1.38 (1.24, 1.52)	0.93 (0.84, 1.04)	0.92 (0.83, 1.03)	0.92 (0.83, 1.03)
Machine assembly	0.90 (0.75, 1.09)	0.91 (0.75, 1.10)	0.90 (0.74, 1.09)	0.90 (0.74, 1.09)
Chemical products	1.17 (0.97, 1.41)	0.93 (0.77, 1.12)	0.93 (0.77, 1.12)	0.93 (0.77, 1.12)
Ceramic products	1.06 (0.84, 1.34)	0.86 (0.68, 1.09)	0.85 (0.67, 1.08)	0.85 (0.67, 1.08)
Electro-mechanic assembly	1.10 (0.91, 1.32)	1.23 (1.01, 1.49)	1.23 (1.02, 1.50)	1.24 (1.02, 1.50)
Transportation machine assembly	1.03 (0.88, 1.20)	0.99 (0.84, 1.15)	0.98 (0.84, 1.15)	0.99 (0.84, 1.15)
Other mechanical assembly	1.27 (0.78, 2.07)	1.33 (0.81, 2.19)	1.34 (0.81, 2.20)	1.34 (0.81, 2.20)
Food manufacturing	1.30 (1.07, 1.58)	1.18 (0.97, 1.44)	1.15 (0.95, 1.41)	1.16 (0.95, 1.41)
Beverage and cigarette	2.12 (1.31, 3.42)	1.42 (0.87, 2.32)	1.35 (0.82, 2.20)	1.35 (0.83, 2.21)
Apparel products	1.49 (1.12, 1.99)	1.03 (0.77, 1.38)	1.04 (0.78, 1.39)	1.04 (0.78, 1.40)
Wooden products	1.48 (1.25, 1.75)	0.99 (0.83, 1.18)	1.00 (0.84, 1.19)	1.00 (0.84, 1.20)
Printing and bookbinding	0.89 (0.62, 1.26)	0.87 (0.61, 1.25)	0.87 (0.61, 1.25)	0.87 (0.61, 1.25)
Rubber and plastic products	1.04 (0.74, 1.45)	1.01 (0.72, 1.42)	1.01 (0.72, 1.41)	1.01 (0.72, 1.42)
Jewelry products	1.59 (1.12, 2.27)	1.36 (0.95, 1.96)	1.37 (0.95, 1.98)	1.37 (0.95, 1.98)
Manufacturing-related workers	1.01 (0.83, 1.23)	1.07 (0.88, 1.31)	1.06 (0.87, 1.30)	1.06 (0.87, 1.30)
Construction machinery operators	1.14 (0.95, 1.36)	1.07 (0.89, 1.28)	1.03 (0.86, 1.23)	1.03 (0.86, 1.24)
Electrical workers	1.20 (1.02, 1.42)	1.13 (0.96, 1.34)	1.11 (0.94, 1.32)	1.12 (0.94, 1.32)
Mine workers	0.94 (0.69, 1.26)	1.02 (0.75, 1.39)	1.04 (0.77, 1.41)	1.04 (0.77, 1.42)
Skeleton construction workers	1.04 (0.83, 1.31)	1.17 (0.93, 1.48)	1.12 (0.89, 1.41)	1.12 (0.89, 1.41)
Construction workers	1.21 (1.08, 1.35)	1.07 (0.96, 1.20)	1.06 (0.94, 1.19)	1.06 (0.94, 1.19)
Civil engineer workers	1.56 (1.35, 1.79)	1.30 (1.12, 1.49)	1.26 (1.09, 1.45)	1.26 (1.09, 1.45)
Cargo workers	1.33 (1.14, 1.54)	1.32 (1.13, 1.53)	1.30 (1.12, 1.51)	1.30 (1.12, 1.52)
Other manual workers	1.39 (1.15, 1.66)	1.46 (1.21, 1.75)	1.44 (1.19, 1.74)	1.44 (1.20, 1.74)
Women				
Professional and engineering				
Researchers	1.76 (0.43, 7.13)	3.81 (0.92, 15.84)	4.08 (0.98, 16.96)	4.09 (0.98, 17.00)
Agriculture, forestry, and fishery engineers	No cases	No cases	No cases	No cases
Food engineers	No cases	No cases	No cases	No cases
Machinery and electrical engineers	0.92 (0.23, 3.73)	2.21 (0.54, 9.04)	2.33 (0.57, 9.55)	2.33 (0.57, 9.57)
Industrial engineers	0.47 (0.07, 3.36)	1.18 (0.16, 8.51)	1.20 (0.16, 8.69)	1.20 (0.16, 8.70)
Other manufacturing engineers	No cases	No cases	No cases	No cases
Architects, civil engineers, surveyors	0.42 (0.06, 2.99)	0.89 (0.12, 6.41)	0.93 (0.13, 6.69)	0.93 (0.13, 6.70)
Data processing engineers	0.26 (0.07, 1.06)	1.10 (0.27, 4.44)	1.17 (0.29, 4.72)	1.17 (0.29, 4.73)
Communication network engineers	No cases	No cases	No cases	No cases
Other engineers	3.52 (0.48, 25.70)	8.39 (1.10, 64.14)	8.45 (1.10, 64.70)	8.45 (1.10, 64.66)
Doctors, dentists, veterinarians, pharmacist	0.96 (0.51, 1.81)	1.14 (0.60, 2.17)	1.14 (0.60, 2.16)	1.13 (0.60, 2.16)
Public health nurses, midwives, nurses	0.99 (0.78, 1.27)	1.01 (0.78, 1.29)	0.99 (0.77, 1.28)	0.98 (0.76, 1.27)
Medical technicians	0.31 (0.11, 0.83)	0.64 (0.24, 1.73)	0.69 (0.26, 1.86)	0.69 (0.26, 1.87)
Other health care workers	0.85 (0.57, 1.27)	0.96 (0.64, 1.44)	0.96 (0.64, 1.44)	0.95 (0.63, 1.43)
Social welfare specialists	0.90 (0.66, 1.24)	1.15 (0.83, 1.58)	1.15 (0.84, 1.59)	1.15 (0.83, 1.59)
Legal workers	No cases	No cases	No cases	No cases
Finance and insurance professionals	0.73 (0.10, 5.20)	0.70 (0.10, 5.12)	0.74 (0.10, 5.40)	0.74 (0.10, 5.41)
Teachers	1.13 (0.85, 1.50)	0.78 (0.59, 1.04)	0.79 (0.59, 1.05)	0.79 (0.59, 1.05)
Workers in religious organisations	2.17 (0.53, 8.83)	0.71 (0.17, 2.98)	0.72 (0.17, 2.99)	0.72 (0.17, 2.99)
Authors, journalists, editors	0.52 (0.07, 3.75)	1.02 (0.14, 7.37)	1.07 (0.15, 7.79)	1.07 (0.15, 7.81)
Artists, designers, photographers, film operators	0.56 (0.18, 1.75)	1.11 (0.35, 3.52)	1.13 (0.36, 3.56)	1.13 (0.36, 3.56)
Musicians, stage designers	No cases	No cases	No cases	No cases
Other specialist professionals	1.19 (0.81, 1.74)	0.92 (0.62, 1.36)	0.93 (0.63, 1.38)	0.93 (0.63, 1.38)
Administrative and managerial workers				
Management staff of government officials	No cases	No cases	No cases	No cases
Officers of organisations	2.45 (1.62, 3.70)	0.93 (0.61, 1.41)	0.90 (0.59, 1.37)	0.90 (0.59, 1.37)
Management staff of organisations	1.11 (0.36, 3.49)	0.88 (0.28, 2.79)	0.84 (0.26, 2.66)	0.84 (0.26, 2.65)
Other managerial workers	5.09 (2.89, 8.97)	2.91 (1.63, 5.21)	2.80 (1.56, 5.02)	2.80 (1.56, 5.01)
Clerical workers				
General clerical workers	reference	reference	reference	reference
Accounting clerks	1.29 (1.00, 1.66)	0.94 (0.72, 1.21)	0.93 (0.72, 1.20)	0.93 (0.72, 1.20)
Production-related clerical workers	1.53 (0.89, 2.62)	1.38 (0.80, 2.38)	1.33 (0.77, 2.30)	1.33 (0.77, 2.30)
Sales clerks	0.70 (0.44, 1.10)	1.20 (0.76, 1.90)	1.21 (0.76, 1.92)	1.21 (0.76, 1.92)
Outdoor service workers	1.28 (0.53, 3.12)	0.76 (0.31, 1.87)	0.75 (0.31, 1.84)	0.75 (0.31, 1.84)
Transport and post clerical workers	1.52 (0.75, 3.08)	1.09 (0.53, 2.23)	1.09 (0.53, 2.23)	1.08 (0.53, 2.23)
Office appliance operators	0.70 (0.29, 1.70)	1.14 (0.46, 2.78)	1.10 (0.45, 2.70)	1.10 (0.45, 2.70)
Sales workers				
Merchandise sales workers	1.75 (1.48, 2.06)	1.13 (0.96, 1.35)	1.11 (0.93, 1.31)	1.10 (0.93, 1.31)
Quasi-sales workers	1.55 (1.18, 2.04)	1.30 (0.99, 1.72)	1.26 (0.95, 1.66)	1.26 (0.95, 1.66)
Service workers				

Domestic support service workers	1.78 (1.21, 2.61)	1.49 (1.01, 2.20)	1.45 (0.98, 2.15)	1.45 (0.98, 2.14)
Care service workers	0.69 (0.40, 1.18)	0.93 (0.54, 1.60)	0.90 (0.52, 1.55)	0.89 (0.51, 1.53)
Domestic hygiene service workers	1.81 (1.38, 2.36)	1.14 (0.87, 1.50)	1.12 (0.86, 1.48)	1.12 (0.86, 1.48)
Food and drink preparatory workers	2.18 (1.79, 2.66)	1.21 (0.99, 1.48)	1.15 (0.94, 1.41)	1.15 (0.94, 1.41)
Customer service workers	1.87 (1.53, 2.28)	1.36 (1.11, 1.67)	1.28 (1.04, 1.57)	1.27 (1.04, 1.57)
Residential facilities management personnel	3.88 (2.29, 6.56)	2.03 (1.18, 3.47)	1.96 (1.14, 3.35)	1.95 (1.14, 3.35)
Other service workers	0.94 (0.46, 1.90)	1.09 (0.53, 2.22)	1.07 (0.52, 2.19)	1.07 (0.52, 2.19)
Security workers				
Self-defense officials	No cases	No cases	No cases	No cases
Judicial police staff	1.11 (0.16, 8.00)	1.93 (0.25, 14.65)	1.79 (0.23, 13.79)	1.78 (0.23, 13.69)
Other public security workers	No cases	No cases	No cases	No cases
Agriculture, forestry, and fishery workers				
Agriculture	7.64 (6.64, 8.78)	1.29 (1.11, 1.51)	1.29 (1.10, 1.50)	1.28 (1.10, 1.50)
Forestry	4.31 (1.58, 11.75)	1.27 (0.46, 3.52)	1.25 (0.45, 3.45)	1.25 (0.45, 3.45)
Fishery	5.38 (3.57, 8.10)	1.52 (1.00, 2.31)	1.49 (0.98, 2.27)	1.49 (0.98, 2.27)
Transport workers				
Railway drivers	No cases	No cases	No cases	No cases
Motor vehicle drivers	1.50 (0.71, 3.20)	1.35 (0.63, 2.89)	1.21 (0.56, 2.60)	1.21 (0.56, 2.60)
Ship and aircraft operators	No cases	No cases	No cases	No cases
Other transport workers	4.41 (1.39, 14.00)	3.16 (0.95, 10.52)	3.18 (0.95, 10.56)	3.17 (0.95, 10.53)
Communication workers	1.18 (0.62, 2.22)	0.75 (0.39, 1.42)	0.75 (0.40, 1.43)	0.75 (0.40, 1.43)
Manufacturing process workers				
Metal products	4.46 (3.49, 5.70)	0.99 (0.76, 1.29)	0.96 (0.74, 1.26)	0.96 (0.74, 1.26)
Machine assembly	2.24 (1.10, 4.55)	1.44 (0.70, 2.96)	1.41 (0.68, 2.90)	1.41 (0.68, 2.90)
Chemical products	2.62 (1.49, 4.59)	1.68 (0.95, 2.97)	1.65 (0.93, 2.92)	1.65 (0.93, 2.92)
Ceramic products	2.18 (1.15, 4.11)	1.04 (0.55, 1.99)	1.02 (0.53, 1.94)	1.02 (0.53, 1.94)
Electro-mechanic assembly	1.90 (1.32, 2.71)	1.33 (0.93, 1.91)	1.34 (0.93, 1.93)	1.34 (0.93, 1.93)
Transportation machine assembly	0.63 (0.20, 1.97)	0.48 (0.15, 1.50)	0.45 (0.14, 1.41)	0.45 (0.14, 1.41)
Other mechanical assembly	3.74 (1.75, 8.00)	3.53 (1.63, 7.68)	3.54 (1.63, 7.70)	3.54 (1.63, 7.70)
Food manufacturing	2.92 (2.38, 3.59)	1.44 (1.16, 1.78)	1.39 (1.13, 1.73)	1.39 (1.13, 1.72)
Beverage and cigarette	4.49 (1.65, 12.25)	1.67 (0.60, 4.69)	1.67 (0.59, 4.70)	1.67 (0.59, 4.69)
Apparel products	3.95 (3.21, 4.87)	1.22 (0.98, 1.51)	1.19 (0.96, 1.48)	1.19 (0.96, 1.48)
Wooden products	4.04 (2.80, 5.83)	1.39 (0.95, 2.02)	1.38 (0.95, 2.01)	1.38 (0.95, 2.01)
Printing and bookbinding	1.71 (0.81, 3.64)	1.08 (0.50, 2.31)	1.05 (0.49, 2.25)	1.05 (0.49, 2.25)
Rubber and plastic products	1.71 (0.84, 3.47)	0.87 (0.43, 1.78)	0.82 (0.40, 1.68)	0.82 (0.40, 1.68)
Jewelry products	3.15 (1.83, 5.43)	0.97 (0.56, 1.69)	0.95 (0.54, 1.65)	0.94 (0.54, 1.65)
Manufacturing-related workers	0.68 (0.28, 1.65)	0.54 (0.22, 1.33)	0.53 (0.22, 1.30)	0.53 (0.22, 1.29)
Construction machinery operators	6.79 (2.12, 21.80)	5.19 (1.55, 17.40)	4.55 (1.35, 15.30)	4.55 (1.35, 15.30)
Electrical workers	No cases	No cases	No cases	No cases
Mine workers	9.55 (2.26, 40.26)	3.01 (0.68, 13.31)	2.54 (0.57, 11.34)	2.54 (0.57, 11.34)
Skeleton construction workers	No cases	No cases	No cases	No cases
Construction workers	4.06 (2.14, 7.70)	1.57 (0.81, 3.03)	1.46 (0.76, 2.82)	1.46 (0.76, 2.82)
Civil engineer workers	3.85 (2.41, 6.17)	1.09 (0.67, 1.77)	1.05 (0.65, 1.70)	1.05 (0.65, 1.70)
Cargo workers	2.08 (1.49, 2.89)	1.33 (0.95, 1.86)	1.30 (0.93, 1.82)	1.30 (0.93, 1.82)
Other manual workers	2.88 (2.39, 3.47)	1.49 (1.23, 1.80)	1.45 (1.20, 1.75)	1.44 (1.19, 1.75)

Model 1: Unadjusted.
Model 2: Adjusted for age, admission date, and hospital.
Model 3: Adjusted for the factors in Model 2 plus smoking, alcohol consumption, and hypertension.
Model 4: Adjusted for the factors in Model 3 plus shift-work.
