# Supplementary material for: A case control study of occupation and cardiovascular disease risk in Japanese men and women
Source: Sci Rep. 2021 Dec 14;11:23983. doi: 10.1038/s41598-021-03410-9 (PMC8671491; doi:10.1038/s41598-021-03410-9)
Supplement: Supplementary file 4 — Supplementary Table S3. [file 41598_2021_3410_MOESM4_ESM.pdf]

S3 Table. Odds ratios for intracerebral hemorrhage by occupations among men and women.

| | Model 1 | Model 2 | Model 3 | Model 4 |
|---|---|---|---|---|
| Men | | | | |
| Professional and engineering | | | | |
| Researchers | 1.40 (0.57, 3.40) | 1.57 (0.64, 3.84) | 1.54 (0.63, 3.79) | 1.55 (0.63, 3.81) |
| Agriculture, forestry, and fishery engineers | 2.15 (1.01, 4.59) | 1.51 (0.70, 3.23) | 1.58 (0.74, 3.40) | 1.60 (0.74, 3.43) |
| Food engineers | 1.46 (0.36, 5.91) | 1.66 (0.41, 6.74) | 1.64 (0.40, 6.71) | 1.65 (0.40, 6.73) |
| Machinery and electrical engineers | 0.82 (0.59, 1.14) | 0.95 (0.69, 1.32) | 0.96 (0.69, 1.34) | 0.96 (0.69, 1.34) |
| Industrial engineers | 0.45 (0.21, 0.95) | 0.50 (0.24, 1.07) | 0.51 (0.24, 1.08) | 0.51 (0.24, 1.08) |
| Other manufacturing engineers | 0.65 (0.21, 2.04) | 0.93 (0.29, 2.91) | 0.96 (0.30, 3.00) | 0.96 (0.30, 3.01) |
| Architects, civil engineers, surveyors | 0.99 (0.74, 1.33) | 1.13 (0.85, 1.52) | 1.13 (0.84, 1.51) | 1.13 (0.84, 1.51) |
| Data processing engineers | 0.50 (0.29, 0.87) | 0.80 (0.46, 1.38) | 0.83 (0.48, 1.44) | 0.82 (0.48, 1.42) |
| Communication network engineers | 0.23 (0.03, 1.67) | 0.31 (0.04, 2.20) | 0.31 (0.04, 2.24) | 0.31 (0.04, 2.23) |
| Other engineers | 0.74 (0.23, 2.31) | 0.92 (0.29, 2.90) | 0.92 (0.29, 2.89) | 0.92 (0.29, 2.89) |
| Doctors, dentists, veterinarians, pharmacists | 0.86 (0.52, 1.43) | 0.87 (0.52, 1.45) | 0.87 (0.52, 1.46) | 0.88 (0.53, 1.47) |
| Public health nurses, midwives, nurses | 0.89 (0.28, 2.78) | 1.05 (0.33, 3.31) | 1.08 (0.34, 3.41) | 1.27 (0.40, 4.00) |
| Medical technicians | 1.00 (0.49, 2.03) | 1.15 (0.56, 2.33) | 1.24 (0.61, 2.53) | 1.25 (0.62, 2.55) |
| Other health care workers | 1.17 (0.58, 2.38) | 1.22 (0.60, 2.48) | 1.29 (0.63, 2.62) | 1.30 (0.64, 2.65) |
| Social welfare specialists | 0.92 (0.43, 1.96) | 1.19 (0.56, 2.55) | 1.27 (0.59, 2.71) | 1.35 (0.63, 2.88) |
| Legal workers | 0.61 (0.15, 2.45) | 0.57 (0.14, 2.30) | 0.58 (0.14, 2.36) | 0.58 (0.14, 2.36) |
| Finance and insurance professionals | 1.36 (0.70, 2.66) | 1.38 (0.71, 2.71) | 1.36 (0.69, 2.66) | 1.35 (0.69, 2.65) |
| Teachers | 1.27 (0.98, 1.63) | 1.16 (0.90, 1.49) | 1.16 (0.90, 1.50) | 1.16 (0.90, 1.50) |
| Workers in religious organisations | 1.34 (0.66, 2.73) | 1.03 (0.51, 2.11) | 1.09 (0.53, 2.22) | 1.09 (0.54, 2.23) |
| Authors, journalists, editors | 0.21 (0.03, 1.50) | 0.24 (0.03, 1.71) | 0.24 (0.03, 1.72) | 0.24 (0.03, 1.73) |
| Artists, designers, photographers, film operators | 1.36 (0.72, 2.58) | 1.52 (0.80, 2.89) | 1.58 (0.83, 2.99) | 1.57 (0.83, 2.98) |
| Musicians, stage designers | No cases | No cases | No cases | No cases |
| Other specialist professionals | 0.85 (0.52, 1.39) | 0.98 (0.60, 1.62) | 1.04 (0.63, 1.70) | 1.04 (0.63, 1.71) |
| Administrative and managerial workers | | | | |
| Management staff of government officials | 1.14 (0.56, 2.32) | 0.97 (0.47, 1.97) | 0.98 (0.48, 1.99) | 0.98 (0.48, 2.00) |
| Officers of organisations | 1.07 (0.84, 1.37) | 0.97 (0.75, 1.24) | 0.96 (0.75, 1.23) | 0.96 (0.75, 1.23) |
| Management staff of organisations | 0.75 (0.53, 1.08) | 0.86 (0.60, 1.23) | 0.84 (0.59, 1.20) | 0.84 (0.59, 1.21) |
| Other managerial workers | 1.36 (0.85, 2.17) | 1.46 (0.91, 2.35) | 1.51 (0.94, 2.42) | 1.51 (0.94, 2.42) |
| Clerical workers | | | | |
| General clerical workers | reference | reference | reference | reference |
| Accounting clerks | 0.91 (0.62, 1.35) | 0.96 (0.65, 1.41) | 0.95 (0.65, 1.41) | 0.96 (0.65, 1.41) |
| Production-related clerical workers | 0.70 (0.43, 1.11) | 0.72 (0.45, 1.15) | 0.71 (0.44, 1.13) | 0.71 (0.44, 1.13) |
| Sales clerks | 1.45 (1.11, 1.91) | 1.62 (1.23, 2.13) | 1.59 (1.21, 2.10) | 1.59 (1.21, 2.09) |
| Outdoor service workers | 1.28 (0.41, 4.03) | 1.16 (0.37, 3.68) | 1.19 (0.37, 3.75) | 1.19 (0.38, 3.78) |
| Transport and post clerical workers | 1.47 (1.00, 2.17) | 1.16 (0.79, 1.72) | 1.18 (0.80, 1.74) | 1.21 (0.82, 1.79) |
| Office appliance operators | No cases | No cases | No cases | No cases |
| Sales workers | | | | |
| Merchandise sales workers | 1.74 (1.43, 2.11) | 1.60 (1.32, 1.94) | 1.64 (1.35, 1.99) | 1.65 (1.36, 2.01) |
| Quasi-sales workers | 1.23 (1.02, 1.47) | 1.36 (1.13, 1.64) | 1.36 (1.13, 1.64) | 1.36 (1.13, 1.64) |
| Service workers | | | | |
| Domestic support service workers | No cases | No cases | No cases | No cases |
| Care service workers | 0.67 (0.21, 2.11) | 1.02 (0.33, 3.22) | 1.09 (0.35, 3.44) | 1.26 (0.40, 3.97) |
| Domestic hygiene service workers | 1.41 (0.92, 2.18) | 1.33 (0.86, 2.05) | 1.36 (0.88, 2.10) | 1.37 (0.88, 2.11) |
| Food and drink preparatory workers | 1.76 (1.36, 2.29) | 1.83 (1.41, 2.37) | 1.86 (1.43, 2.42) | 1.88 (1.45, 2.45) |
| Customer service workers | 1.31 (0.90, 1.90) | 1.44 (0.99, 2.09) | 1.52 (1.05, 2.21) | 1.56 (1.07, 2.27) |
| Residential facilities management personnel | 1.33 (0.66, 2.70) | 1.47 (0.72, 2.99) | 1.47 (0.72, 3.01) | 1.52 (0.75, 3.10) |
| Other service workers | 1.60 (0.82, 3.12) | 1.75 (0.89, 3.44) | 1.83 (0.93, 3.60) | 1.87 (0.95, 3.67) |
| Security workers | | | | |
| Self-defense officials | 0.66 (0.38, 1.13) | 0.56 (0.33, 0.97) | 0.58 (0.33, 0.99) | 0.59 (0.34, 1.01) |
| Judicial police staff | 0.94 (0.57, 1.54) | 1.06 (0.65, 1.74) | 1.06 (0.65, 1.74) | 1.13 (0.69, 1.87) |
| Other public security workers | 1.27 (0.90, 1.81) | 1.32 (0.93, 1.87) | 1.32 (0.93, 1.88) | 1.46 (1.03, 2.08) |
| Agriculture, forestry, and fishery workers | | | | |
| Agriculture | 2.44 (2.04, 2.93) | 1.33 (1.10, 1.60) | 1.42 (1.18, 1.72) | 1.43 (1.18, 1.72) |
| Forestry | 1.94 (1.08, 3.48) | 1.22 (0.68, 2.19) | 1.28 (0.71, 2.31) | 1.28 (0.71, 2.31) |
| Fishery | 2.13 (1.63, 2.80) | 1.57 (1.19, 2.08) | 1.66 (1.26, 2.19) | 1.66 (1.26, 2.20) |
| Transport workers | | | | |
| Railway drivers | 1.72 (0.98, 3.02) | 1.40 (0.79, 2.46) | 1.39 (0.79, 2.45) | 1.44 (0.82, 2.54) |
| Motor vehicle drivers | 1.49 (1.24, 1.79) | 1.39 (1.15, 1.67) | 1.39 (1.15, 1.67) | 1.41 (1.18, 1.70) |
| Ship and aircraft operators | 1.96 (1.23, 3.14) | 1.62 (1.01, 2.60) | 1.68 (1.05, 2.70) | 1.74 (1.08, 2.80) |
| Other transport workers | 1.42 (0.95, 2.12) | 1.21 (0.81, 1.81) | 1.25 (0.83, 1.87) | 1.28 (0.85, 1.92) |
| Communication workers | 1.11 (0.41, 3.01) | 1.11 (0.41, 2.99) | 1.13 (0.42, 3.06) | 1.15 (0.42, 3.12) |
| Manufacturing process workers | | | | |

| | | | | |
|---|---|---|---|---|
| Metal products | 1.69 (1.42, 2.01) | 1.24 (1.04, 1.48) | 1.28 (1.07, 1.53) | 1.30 (1.08, 1.55) |
| Machine assembly | 1.49 (1.13, 1.97) | 1.44 (1.09, 1.90) | 1.46 (1.10, 1.94) | 1.47 (1.11, 1.95) |
| Chemical products | 1.29 (0.94, 1.76) | 1.06 (0.77, 1.46) | 1.08 (0.79, 1.49) | 1.13 (0.82, 1.55) |
| Ceramic products | 1.16 (0.78, 1.74) | 1.04 (0.70, 1.56) | 1.06 (0.71, 1.59) | 1.08 (0.72, 1.61) |
| Electro-mechanic assembly | 1.06 (0.75, 1.49) | 1.05 (0.74, 1.48) | 1.08 (0.76, 1.53) | 1.12 (0.79, 1.58) |
| Transportation machine assembly | 1.10 (0.84, 1.44) | 1.03 (0.78, 1.35) | 1.06 (0.81, 1.39) | 1.08 (0.82, 1.41) |
| Other mechanical assembly | 0.73 (0.23, 2.28) | 0.72 (0.23, 2.27) | 0.74 (0.23, 2.32) | 0.74 (0.24, 2.34) |
| Food manufacturing | 1.79 (1.32, 2.42) | 1.59 (1.17, 2.15) | 1.63 (1.20, 2.21) | 1.65 (1.22, 2.24) |
| Beverage and cigarette | 2.30 (1.01, 5.20) | 1.54 (0.68, 3.51) | 1.47 (0.64, 3.35) | 1.50 (0.66, 3.42) |
| Apparel products | 2.06 (1.32, 3.19) | 1.52 (0.97, 2.37) | 1.61 (1.03, 2.52) | 1.63 (1.05, 2.55) |
| Wooden products | 1.72 (1.29, 2.29) | 1.18 (0.88, 1.58) | 1.22 (0.91, 1.63) | 1.24 (0.93, 1.66) |
| Printing and bookbinding | 1.40 (0.84, 2.33) | 1.41 (0.84, 2.35) | 1.45 (0.87, 2.42) | 1.47 (0.88, 2.46) |
| Rubber and plastic products | 1.37 (0.81, 2.31) | 1.36 (0.80, 2.31) | 1.40 (0.82, 2.37) | 1.43 (0.85, 2.43) |
| Jewelry products | 0.78 (0.32, 1.91) | 0.77 (0.32, 1.88) | 0.82 (0.33, 1.99) | 0.83 (0.34, 2.02) |
| Manufacturing-related workers | 1.36 (0.99, 1.86) | 1.31 (0.96, 1.79) | 1.37 (1.00, 1.88) | 1.37 (1.00, 1.88) |
| Construction machinery operators | 0.90 (0.63, 1.27) | 0.82 (0.58, 1.17) | 0.81 (0.57, 1.16) | 0.83 (0.58, 1.18) |
| Electrical workers | 1.54 (1.18, 2.00) | 1.43 (1.10, 1.87) | 1.45 (1.11, 1.89) | 1.45 (1.11, 1.90) |
| Mine workers | 0.91 (0.53, 1.56) | 1.25 (0.72, 2.17) | 1.33 (0.77, 2.30) | 1.34 (0.78, 2.33) |
| Skeleton construction workers | 0.68 (0.41, 1.11) | 0.68 (0.41, 1.11) | 0.71 (0.43, 1.16) | 0.70 (0.43, 1.15) |
| Construction workers | 1.87 (1.56, 2.24) | 1.63 (1.36, 1.95) | 1.69 (1.41, 2.03) | 1.69 (1.41, 2.02) |
| Civil engineer workers | 1.62 (1.27, 2.07) | 1.35 (1.05, 1.72) | 1.40 (1.09, 1.79) | 1.39 (1.09, 1.78) |
| Cargo workers | 1.46 (1.13, 1.89) | 1.39 (1.07, 1.80) | 1.44 (1.11, 1.86) | 1.45 (1.12, 1.88) |
| Other manual workers | 1.74 (1.29, 2.34) | 1.72 (1.27, 2.32) | 1.80 (1.33, 2.43) | 1.81 (1.34, 2.45) |
| Women | | | | |
| Professional and engineering | | | | |
| Researchers | 1.47 (0.20, 10.54) | 2.47 (0.34, 17.90) | 2.72 (0.37, 19.72) | 2.72 (0.37, 19.74) |
| Agriculture, forestry, and fishery engineers | No cases | No cases | No cases | No cases |
| Food engineers | 2.03 (0.28, 14.60) | 3.46 (0.47, 25.71) | 3.85 (0.52, 28.63) | 3.85 (0.52, 28.68) |
| Machinery and electrical engineers | No cases | No cases | No cases | No cases |
| Industrial engineers | 0.79 (0.11, 5.63) | 1.54 (0.21, 11.10) | 1.55 (0.21, 11.23) | 1.55 (0.21, 11.24) |
| Other manufacturing engineers | No cases | No cases | No cases | No cases |
| Architects, civil engineers, surveyors | 0.70 (0.10, 5.01) | 1.18 (0.16, 8.53) | 1.23 (0.17, 8.86) | 1.23 (0.17, 8.88) |
| Data processing engineers | No cases | No cases | No cases | No cases |
| Communication network engineers | No cases | No cases | No cases | No cases |
| Other engineers | No cases | No cases | No cases | No cases |
| Doctors, dentists, veterinarians, pharmacist | 0.80 (0.33, 1.96) | 0.95 (0.39, 2.33) | 0.95 (0.39, 2.32) | 0.95 (0.39, 2.32) |
| Public health nurses, midwives, nurses | 0.68 (0.46, 0.98) | 0.65 (0.45, 0.95) | 0.65 (0.45, 0.95) | 0.65 (0.44, 0.95) |
| Medical technicians | 0.51 (0.19, 1.39) | 0.81 (0.30, 2.20) | 0.88 (0.33, 2.39) | 0.88 (0.33, 2.39) |
| Other health care workers | 0.82 (0.48, 1.39) | 0.86 (0.51, 1.47) | 0.87 (0.51, 1.47) | 0.86 (0.51, 1.47) |
| Social welfare specialists | 0.72 (0.46, 1.13) | 0.77 (0.49, 1.22) | 0.78 (0.49, 1.23) | 0.78 (0.49, 1.23) |
| Legal workers | No cases | No cases | No cases | No cases |
| Finance and insurance professionals | No cases | No cases | No cases | No cases |
| Teachers | 1.33 (0.95, 1.88) | 1.03 (0.73, 1.46) | 1.04 (0.74, 1.47) | 1.04 (0.74, 1.47) |
| Workers in religious organisations | 5.44 (1.71, 17.27) | 2.58 (0.80, 8.37) | 2.60 (0.80, 8.43) | 2.60 (0.80, 8.42) |
| Authors, journalists, editors | No cases | No cases | No cases | No cases |
| Artists, designers, photographers, film operators | 0.62 (0.15, 2.51) | 1.04 (0.26, 4.24) | 1.07 (0.26, 4.33) | 1.07 (0.26, 4.34) |
| Musicians, stage designers | 2.09 (0.52, 8.50) | 3.55 (0.86, 14.71) | 3.79 (0.91, 15.71) | 3.79 (0.91, 15.72) |
| Other specialist professionals | 1.23 (0.75, 2.00) | 1.10 (0.67, 1.79) | 1.12 (0.69, 1.83) | 1.12 (0.69, 1.83) |
| Administrative and managerial workers | | | | |
| Management staff of government officials | No cases | No cases | No cases | No cases |
| Officers of organisations | 3.11 (1.93, 5.01) | 1.40 (0.86, 2.28) | 1.38 (0.85, 2.25) | 1.38 (0.85, 2.25) |
| Management staff of organisations | 2.48 (0.91, 6.71) | 2.16 (0.79, 5.91) | 2.04 (0.74, 5.59) | 2.04 (0.74, 5.59) |
| Other managerial workers | 1.96 (0.62, 6.17) | 1.58 (0.50, 5.03) | 1.48 (0.46, 4.73) | 1.48 (0.46, 4.73) |
| Clerical workers | | | | |
| General clerical workers | | | | |
| Accounting clerks | 1.15 (0.82, 1.61) | 0.89 (0.63, 1.26) | 0.89 (0.63, 1.25) | 0.89 (0.63, 1.25) |
| Production-related clerical workers | 0.55 (0.17, 1.71) | 0.50 (0.16, 1.56) | 0.48 (0.15, 1.52) | 0.48 (0.15, 1.52) |
| Sales clerks | 0.47 (0.23, 0.95) | 0.63 (0.31, 1.28) | 0.64 (0.31, 1.30) | 0.64 (0.31, 1.30) |
| Outdoor service workers | 1.28 (0.41, 4.03) | 0.90 (0.28, 2.83) | 0.89 (0.28, 2.80) | 0.88 (0.28, 2.80) |
| Transport and post clerical workers | 0.95 (0.30, 2.98) | 0.79 (0.25, 2.49) | 0.78 (0.25, 2.48) | 0.78 (0.25, 2.47) |
| Office appliance operators | 0.23 (0.03, 1.67) | 0.32 (0.05, 2.31) | 0.31 (0.04, 2.25) | 0.31 (0.04, 2.25) |
| Sales workers | | | | |
| Merchandise sales workers | 1.67 (1.34, 2.07) | 1.24 (1.00, 1.55) | 1.23 (0.99, 1.54) | 1.23 (0.99, 1.54) |
| Quasi-sales workers | 1.51 (1.06, 2.16) | 1.33 (0.93, 1.89) | 1.31 (0.92, 1.87) | 1.31 (0.92, 1.87) |
| Service workers | | | | |

| | | | | |
|---|---|---|---|---|
| Domestic support service workers | 1.23 (0.68, 2.21) | 1.03 (0.57, 1.85) | 1.02 (0.57, 1.84) | 1.02 (0.56, 1.84) |
| Care service workers | 0.99 (0.55, 1.78) | 1.27 (0.70, 2.30) | 1.27 (0.70, 2.30) | 1.26 (0.69, 2.28) |
| Domestic hygiene service workers | 1.48 (1.02, 2.16) | 1.06 (0.72, 1.54) | 1.07 (0.73, 1.56) | 1.07 (0.73, 1.56) |
| Food and drink preparatory workers | 2.00 (1.54, 2.60) | 1.25 (0.96, 1.64) | 1.20 (0.92, 1.56) | 1.20 (0.92, 1.56) |
| Customer service workers | 1.38 (1.03, 1.84) | 1.05 (0.78, 1.40) | 0.99 (0.74, 1.33) | 0.99 (0.74, 1.33) |
| Residential facilities management personnel | 2.16 (0.88, 5.27) | 1.24 (0.50, 3.05) | 1.23 (0.50, 3.03) | 1.23 (0.50, 3.03) |
| Other service workers | 0.78 (0.29, 2.12) | 0.87 (0.32, 2.36) | 0.87 (0.32, 2.37) | 0.87 (0.32, 2.37) |
| Security workers | | | | |
| Self-defense officials | No cases | No cases | No cases | No cases |
| Judicial police staff | No cases | No cases | No cases | No cases |
| Other public security workers | 1.10 (0.15, 7.92) | 1.08 (0.15, 7.83) | 1.03 (0.14, 7.44) | 1.03 (0.14, 7.42) |
| Agriculture, forestry, and fishery workers | | | | |
| Agriculture | 4.36 (3.58, 5.31) | 1.02 (0.82, 1.27) | 1.05 (0.84, 1.30) | 1.04 (0.84, 1.30) |
| Forestry | 1.80 (0.25, 12.94) | 0.58 (0.08, 4.22) | 0.55 (0.08, 4.02) | 0.55 (0.08, 4.03) |
| Fishery | 1.73 (0.71, 4.21) | 0.58 (0.24, 1.43) | 0.59 (0.24, 1.44) | 0.59 (0.24, 1.44) |
| Transport workers | | | | |
| Railway drivers | No cases | No cases | No cases | No cases |
| Motor vehicle drivers | 2.15 (0.95, 4.87) | 1.90 (0.83, 4.34) | 1.75 (0.76, 4.00) | 1.75 (0.76, 3.99) |
| Ship and aircraft operators | No cases | No cases | No cases | No cases |
| Other transport workers | No cases | No cases | No cases | No cases |
| Communication workers | 0.98 (0.40, 2.39) | 0.73 (0.30, 1.78) | 0.74 (0.30, 1.82) | 0.74 (0.30, 1.81) |
| Manufacturing process workers | | | | |
| Metal products | 3.59 (2.54, 5.06) | 1.14 (0.79, 1.65) | 1.12 (0.77, 1.62) | 1.12 (0.77, 1.62) |
| Machine assembly | 0.93 (0.23, 3.78) | 0.68 (0.17, 2.78) | 0.68 (0.17, 2.77) | 0.68 (0.17, 2.77) |
| Chemical products | 1.01 (0.32, 3.17) | 0.74 (0.24, 2.34) | 0.73 (0.23, 2.32) | 0.73 (0.23, 2.32) |
| Ceramic products | 1.82 (0.74, 4.43) | 0.99 (0.40, 2.44) | 0.97 (0.39, 2.40) | 0.97 (0.39, 2.40) |
| Electro-mechanic assembly | 1.49 (0.89, 2.49) | 1.04 (0.62, 1.75) | 1.05 (0.62, 1.76) | 1.04 (0.62, 1.75) |
| Transportation machine assembly | 1.76 (0.72, 4.29) | 1.40 (0.57, 3.45) | 1.35 (0.55, 3.32) | 1.35 (0.55, 3.32) |
| Other mechanical assembly | 1.78 (0.44, 7.23) | 1.54 (0.38, 6.27) | 1.58 (0.39, 6.44) | 1.58 (0.39, 6.44) |
| Food manufacturing | 2.35 (1.77, 3.13) | 1.24 (0.93, 1.66) | 1.23 (0.92, 1.65) | 1.23 (0.92, 1.65) |
| Beverage and cigarette | 1.87 (0.26, 13.49) | 0.74 (0.10, 5.40) | 0.74 (0.10, 5.45) | 0.74 (0.10, 5.45) |
| Apparel products | 1.84 (1.28, 2.62) | 0.74 (0.52, 1.07) | 0.73 (0.51, 1.06) | 0.73 (0.51, 1.05) |
| Wooden products | 3.27 (1.95, 5.48) | 1.38 (0.82, 2.32) | 1.39 (0.82, 2.35) | 1.39 (0.82, 2.35) |
| Printing and bookbinding | 0.82 (0.20, 3.30) | 0.59 (0.15, 2.39) | 0.57 (0.14, 2.31) | 0.57 (0.14, 2.31) |
| Rubber and plastic products | 1.78 (0.73, 4.36) | 1.13 (0.46, 2.79) | 1.07 (0.44, 2.64) | 1.07 (0.44, 2.64) |
| Jewelry products | 2.26 (1.00, 5.11) | 1.17 (0.51, 2.69) | 1.17 (0.51, 2.67) | 1.16 (0.51, 2.67) |
| Manufacturing-related workers | 1.37 (0.60, 3.09) | 1.16 (0.51, 2.64) | 1.15 (0.50, 2.62) | 1.15 (0.50, 2.61) |
| Construction machinery operators | No cases | No cases | No cases | No cases |
| Electrical workers | No cases | No cases | No cases | No cases |
| Mine workers | 7.96 (1.08, 58.87) | 3.12 (0.40, 24.45) | 2.69 (0.34, 21.29) | 2.69 (0.34, 21.29) |
| Skeleton construction workers | No cases | No cases | No cases | No cases |
| Construction workers | 5.42 (2.65, 11.11) | 2.58 (1.25, 5.35) | 2.51 (1.21, 5.22) | 2.51 (1.21, 5.22) |
| Civil engineer workers | 3.72 (2.01, 6.88) | 1.41 (0.75, 2.63) | 1.39 (0.74, 2.61) | 1.39 (0.74, 2.61) |
| Cargo workers | 2.03 (1.32, 3.12) | 1.46 (0.95, 2.25) | 1.43 (0.93, 2.21) | 1.43 (0.93, 2.21) |
| Other manual workers | 2.47 (1.92, 3.17) | 1.47 (1.14, 1.90) | 1.46 (1.13, 1.88) | 1.45 (1.12, 1.88) |

Model 1: Unadjusted.
Model 2: Adjusted for age, admission date, and hospital.
Model 3: Adjusted for the factors in Model 2 plus smoking, alcohol consumption, and hypertension.
Model 4: Adjusted for the factors in Model 3 plus shift-work.
